# Acknowledgement to Reviewers of *Medical Sciences* in 2017

**DOI:** 10.3390/medsci6010007

**Published:** 2018-01-19

**Authors:** Medical Sciences Editorial Office

**Affiliations:** MDPI AG, St. Alban-Anlage 66, 4052 Basel, Switzerland

Peer review is an essential part in the publication process, ensuring that *Medical Sciences* maintains high quality standards for its published papers. In 2017, a total of 37 papers were published in the journal. Thanks to the cooperation of our reviewers, the median time to first decision was 18 days and the median time to publication was 49 days. The editors would like to express their sincere gratitude to the following reviewers for their time and dedication in 2017:
Arisan, Elif DamlaKirchengast, SylviaBahtiyar, Mert OzanKitamura, TakanoriBaroni, ArgelindaKlettner, AlexaBeninati, SimoneKozarek, RichardBernabe-Ortiz, AntonioLakhkar, AnandBettaieb, AliLalatta, FaustinaBisio, AlessandraLevenson, JessicaBlum, Jason L.Li, DapengBrewster, Lizzy MLiu, Guang-YawBrun, Jean-FrédéricManary, Mark J.Bucolo, ClaudioMedina-Ramírez, IlianaBukowski, HenrykMeijles, DanielCalabrese, OlgaMill, JoseCampuzano, OscarMirza, ShaperCardenas Zuniga, RobertoMizuno, KohCardozo, EdenMohammed, AltafCartland, SianMoisés, JorgeCasero, RobertMonif, MasturaCatry, BoudewijnMontalvo González, EfigeniaCeccato, AdriánMoss, JonCervelli, ManuelaMurai, NoriyukiChen, SuzieNana, Andre W.Chim, StephenNancy, AnnChlebowski, Rowan T.Naumann, David NCoppola, GianlucaNekludov, MichaelCresci, GailNowotarski, ShannonCui, ZhibinOberheim, BushCunningham, PatrickOmote, HiroshiDagradi, FedericaPapi, RiginiDatta Chaudhuri, AmritaPark, JoshuaDavis, Thomas P.Parr, DavidDe La Rosa Carrillo, DavidPegg, Anthony EDen Haan, JurrePirillo, AngelaDevaraj, AishwaryaProsseda, GianniDidonna, AlessandroPucciarelli, SandraDietrich, Johannes W.Rademacher, JessicaDoan, NinhRamtekkar, UjjwalFeith, DavidRathinasabapathy, AnandharajanFeng, YuRedmer, TorbenFlynn, AndreaRhee, Joon HaengFredriksson-Larsson, UllaRosales-Mayor, EdmundoGarcia-Fruitós, ElenaRosenfeld, SteveGarcia-Velasco, JuanRostasy, KevinGastañaduy, PaulShteinberg, MichalGeethakumari, Praveen RamakrishnanSmith, BradfordGeorge, CindySong, SuGilmour, SusanSpencer, DrewGobert, Alain P.Stecca, BarbaraGotsbacher, MichaelStenzelius, KarinGursel, MaydaStrack, PeterHait, NitaiSubramaniam, DharmalingamHalasa, YaraTakeda, MasakiHao, LeiTorrejón, RafaelHassan Hassan M. A.Tringali, AndreaHayes, LawrenceTsukahara, TomohideHayes, LawrenceUlivieri, CristinaHeeschen, ChristopherVan Zanten, MalouHoudebine, Louis-MarieVermeulen, LouisIgarashi, KazueiVillarini, AnnaInamura, KentaroWang, Peng-HuiIvanov, Alexander VWard, LeighJain, NitinWeeraratna, AshaniJakovljevic, MihajloWilson, RobertJenkins, Jill A.Withers, MellissaJeong, Seok HoonWolansky, Marcelo JavierJimenez-Escrig, AdrianoWoster, PatrickJin, LeiXiang, YuKabra, RitaYap, Desmond Y. H.Kalimeri, KyriakiYou, XiaonaKamijo-Ikemori, AtsukoZuhorn, Inge S.Kim, Yumi


